# Shamba Maisha: A pilot study assessing impacts of a micro-irrigation intervention on the health and economic wellbeing of HIV patients

**DOI:** 10.1186/1471-2458-10-245

**Published:** 2010-05-11

**Authors:** Jay A Pandit, Nicole Sirotin, Robin Tittle, Elijah Onjolo, Elizabeth A Bukusi, Craig R Cohen

**Affiliations:** 1 Department of Obstetrics, Gynecology and Reproductive Sciences, University of California San Francisco School of Medicine, USA; 2 Division of Global Public Health, Department of Medicine, University of California San Diego School of Medicine, USA; 3 Department of Medicine, Albert Einstein College of Medicine/Montefiore Medical Center, USA; 4 Centre for Microbiology Research, Kenya Medical Research Institute, Kenya

## Abstract

**Background:**

HIV/AIDS negatively impacts poverty alleviation and food security, which reciprocally hinder the rapid scale up and effectiveness of HIV care programs. Nyanza province has the highest HIV prevalence (15.3%), and is the third highest contributor (2.4 million people) to rural poverty in Kenya. Thus, we tested the feasibility of providing a micro-irrigation pump to HIV-positive farmers in order to evaluate its impact on health and economic advancement among HIV-positive patients and their families.

**Methods:**

Thirty HIV-positive patients enrolled in the Family AIDS Care and Education Services (FACES) program in Kisumu, Kenya were provided a micro-financed loan to receive an irrigation pump and farming guidance from KickStart, the developer of the pump. Economic data, CD4 counts, household health and loan repayment history were collected 12 months after the pumps were distributed.

**Results:**

Mean annual family income increased by $1,332 over baseline. CD4 counts did not change significantly. Though income increased, only three (10%) participants had paid off more than a quarter of the loan.

**Conclusions:**

We demonstrated the feasibility of an income-generating micro-irrigation intervention among HIV-positive patients and the collection of health and economic data. While family income improved significantly, loan repayment rates were low- likely complicated by the drought that occurred in Kenya during the intervention period.

## Background

The United Nations Millennium Development Goals (MDG) highlight eradicating extreme poverty and hunger and combating HIV/AIDS as two main priorities for the global community [1]. Studies have documented the increased vulnerability to HIV/AIDS that results from food insecurity [2], the negative impacts of HIV/AIDS on poverty alleviation and food security [3] and the reciprocal negative effect of food insufficiency on the health and behaviors of HIV positive individuals [4,5]. Accordingly, the World Health Organization, UNAIDS, the World Food Program (WFP) and other international organizations have emphasized the integration of food and HIV/AIDS programming activities where possible [6,7]. One important piece of poverty and hunger eradication is the development of independent farming capabilities for rural populations [8].

In Kenya, agriculture is the leading economic sector, accounting for 25% of the gross domestic product (GDP) and employing 61% of working Kenyans [9]. However, both a shortage of accessible water and the burden of HIV on some communities pose challenges to successful subsistence farming. Nyanza Province has the highest (15.3%) HIV prevalence in the country [10,11]. The combination of a heavy dependence on an unstable agricultural sector and such high HIV prevalence makes residents of Nyanza Province particularly vulnerable to severe poverty and malnutrition.

Recognizing the need for improved agricultural tools for poor farmers in Kenya, KickStart, an international non-governmental organization, developed a low-cost micro-irrigation pump which is purchased by local entrepreneurs and used to establish new, small agricultural businesses. These pumps allow users to irrigate their crops year-round and not depend solely on seasonal rainfall. Being able to irrigate crops during the dry season allows pump owners to take advantage of the higher crop prices in the marketplace. Successful models of micro-irrigation in India and Nepal have been found to increase crop yields by up to 200% and reduce water consumption by 40-70% [12], in addition to increasing income and household food security. Since 1996 KickStart has been one of the leaders in micro-irrigation technologies through the development and sales of its manually operated "MoneyMaker" pumps. "Farmerpreneurs" are increasing their incomes by as much as ten-fold and making as much as $5400 in profit per year, transforming subsistence farms into highly profitable enterprises [13]. Although, the agricultural and financial benefits of the micro-irrigation technology have been demonstrated, little is known about its effect on health outcomes in general, and in particular, farmers living with HIV.

Micro-finance loans with financial advising have been used throughout Africa to support entrepreneurs who do not have access to mainstream financial institutions [14]. While microfinance has been found to significantly improve several health outcomes [15,16] its limitations have been underscored with particular reference to it as a stand-alone strategy. As such, recent research has recommended a merger between microfinance and other livelihood approaches to maximize health and poverty reduction outcomes [17,18]. Thus less is known about the health and income effects on HIV-positive farmers of a merged microfinance and agricultural technology program. Our pilot study evaluated the feasibility of assessing heath and economic impacts of providing HIV-positive farmers enrolled in an HIV care and treatment program a microfinance loan in-kind to receive farming commodities including the "MoneyMaker" pump.

## Methods

### Population and Setting

Family AIDS Care and Education Services (FACES) is an HIV care and treatment program in Kenya managed by the Kenya Medical Research Institute (KEMRI) and the University of California, San Francisco (UCSF), funded through the US President's Emergency Plan for AIDS Relief (PEPFAR). Since 2004, FACES has offered services in the diagnosis and treatment of HIV and related illnesses in addition to counseling, support groups, and peer education free of charge. In 2005, FACES expanded to Kisumu (the 3^rd ^largest city in Kenya located on Lake Victoria) at the Lumumba Health Center, Kisumu's busiest maternal health facility.

Between February 2007 and July 2007, community health counselors from the FACES program recruited thirty FACES patients from the Lumumba Health Center in Kisumu, Kenya. Eligibility requirements to receive a loan included participation in FACES treatment program, ability to make a down payment of 600 Kenya shillings (Ksh), approximately US $8, and access to land with an adjacent supply of surface water for farming.

### Intervention

The KEMRI Ethical Review Committee and UCSF Committee on Human Research reviewed and approved the study protocol. Eligible participants were counseled on the details of the project and asked to sign a commitment contract. In addition, all participants signed a written informed consent form. After commitment, the farmer received a local purchase order (LPO) to obtain their inputs from the Mwanga Agrovet (a local store). The inputs included: KickStart's MoneyMaker Hip Pump, a hose pipe, an inlet pipe, seeds, fertilizer, and pesticides. Farmers were able to choose seeds from an assorted range of different vegetable crops to plant. These crops were recommended by other farmers and support group members as successful in the area and approved by FACES and KickStart field experts.

### Loan Scheme

The in-kind loan was for approximately 6600 Ksh (US$95). The farmers agreed to repay the loans within a year, during which we anticipated the farmers would have two crop cycles. They were asked to make a down payment of at least 600 Ksh, or approximately 10% of the loan. Though the recruitment communication was primarily with the HIV positive subject, the economic outcome measure was total family income. Thus, it was anticipated that the farming activities would be carried out by the subjects and their families.

### Training

The project coordinator and FACES counselors conducted initial site visits to train farmers and their families on using the pump, preparing their fields, and planting their seed beds. Thereafter informal quarterly site visits were conducted to ensure proper pump use, follow progress and incomes as well as to provide advice on marketing and sales of vegetables.

### Focus Group Discussion

At the conclusion of the study, eighteen farmers participated in three focus groups, sharing the benefits and challenges of the project and their experiences with loan repayment. Focus groups were divided by gender to allow for consideration of unique experiences between men and women. The groups were facilitated by trained staff.

### Data Collection

Trained staff of KickStart's Monitoring and Evaluation (M&E) Unit (Kihia 2003) and staff from FACES interviewed participants at baseline and 12 months after provision of the loan. The questionnaire, administered by the KickStart M&E Unit, evaluated household income, assets, type of housing, land ownership, total irrigated acres of land, types of crops planted, irrigated income, total family income, expenditures and food security at baseline and 12 months. A household survey administered by FACES staff at 12 months collected health and nutrition data of household members, including illnesses in the family during the past month, children's missed school days for illness, dietary composition, basic hygiene and household water sources. Clinical health data were also assessed at baseline and at 12 months using the FACES patient database. These indicators included body mass index (BMI) and CD4 T-cell counts.

### Statistical Analysis

Data was entered into Excel 2003 (Microsoft, Renton, WA) and SAS version 9.2 (Cary, NC) was used for statistical analysis. Data were analyzed using Chi-squared test or Fisher's exact test, as appropriate, and continuous variables were analyzed using the Student's T-test. To examine the change in BMI and CD4 count from baseline to 12 months, the Cochrane's test was used. Qualitative data was coded and analyzed by one of the authors (RT).

## Results

### Demographic/Socioeconomic Factors

Thirty participants were recruited from four districts in Nyanza Province; 22 from Kisumu (76%), 4 from Nyando (14%), 2 from Rachuonyo (7%) and 1 from Suba (3%). One participant, later found to be HIV-negative, was subsequently exited from the study. Demographic characteristics of the 22 male (20 married, 2 widowers) and 8 female farmers (4 married, 4 widows) that were initially recruited are shown in Table 1. Most participants (80%) had family sizes greater than three, had only a primary school education (83%), and reported farming as their main occupation and primary source of income (73%). Fifty eight percent of the participants had monthly incomes of less than $10 (approximately a seventh of the monthly Gross National Index (GNI) reported by the World Bank in 2007) and nearly all reported that either food or school fees were their main household expenses.

**Table 1:** Demographic Data of HIV+ farmers enrolled in the Shamba Maisha project in Kisumu, Kenya.

N = 29*	**Number (%)**
**Age years, mean ± SD**	41 ± 9.7
25-35 years	12 (42%)
36-45 years	7 (24%)
46-55 years	7 (24%)
56-65 years	3 (10%)
**Sex**	
Male	22 (75%)
Female	7 (25%)
**Family Size, mean ± SD**	5.2 ± 2.3
0-1	0
2-5	15 (51%)
>5	14 (49%)
**Education Level**	
Primary School	24 (83%)
Secondary School	3 (10%)
College	2 (7%)
**Main Occupation**	
Farming	21 (73%)
Business	3 (10%)
Waged employment	5 (17%)
**Monthly Income (US Dollars)**,	
$0 - $5	8 (27%)
$5 - $10	9 (31%)
>$10	12 (42%)
**Main Household Expenses**	
Food	21 (72%)
School fees	6 (21%)
Others	2 (7%)
**Type of Roof**	
Iron Sheet	27 (93%)
Grass thatched	2 (7%)
**Type of Walls**	
Mud	21 (72%)
Brick	6 (20%)
Iron Sheet	2 (7%)

* Thirty participants were recruited, but one was found to be HIV negative and subsequently exited from the study.

### Health Parameters

There were no significant differences between baseline and one year follow up in patient's BMI (Table 2). All three patients with CD4 counts below 200 cells/mcL at baseline had CD4 counts greater than 200 cells/mcL after 12 months. Of note, 80% of participants were receiving antiretroviral therapy (ART) throughout the study period. Of the six participants that were not on ART at baseline, two were started on ART by the 12-month time point.

**Table 2:** BMI and CD4 count at baseline and for HIV+ farmers enrolled in the Shamba Maisha program in Kisumu, Kenya.

	Baseline	12 months	p-Value
**BMI**			0.17
< 20	11 (38%)	10 (35%)	
20 - 25	15 (52%)	16 (55%)	
>25	3 (10%)	3 (10%)	
			
**CD4 cells/mcL**			0.96
<200	3 (10%)	0	
200 - 500	20 (69%)	20 (69%)	
>500	6 (21%)	9 (41%)	

### Household Data

Household data was collected at the 12-month follow-up visit (Table 3). Forty-nine percent of the farmers had a household size greater than five, with an average of three child dependents. Almost one fifth of the households reported having gone an average of 1.4 days without food during the month before the interview, over one third reported that they did not boil their water before use, and 15% of all children living in these households reported missing school due to illness in the past month.

**Table 3:** Household health indicator data of HIV+ farmers enrolled in Shamba Maisha and their households at 12 months in Kisumu, Kenya.

Variable	Number (%)
**Child Dependants per household, mean ± SD**	3.0 ± 1.7
**Total child dependents**	79
**Diseases in the past month**	N = 167*
Fever	33 (20%)
Respiratory illness	35 (21%)
Diarrhea	6 (3%)
Malaria	43 (26%)
Other illness	15 (9%)
Missed school	25 (15%)
**Source of Food for household**	**N = 29**
Farm	16 (55%)
Purchase	13 (45%)
**Food Insecurity for household**	**N = 29**
Food By Prescription	3 (10%)
Boil water	19 (65%)
Days with no food in past month	5 (17%)
If yes how many days (mean)	1.4
Average meat meals/wk	2.5
Average vegetable meals/wk	10.4

* The total number of people including enrolled subject and those designated as part of their family unit at baseline.

### Economic Data

At the 12-month follow-up visit the mean annual family income increased significantly by $1,332 (Range: -$267 to $4825, p < 0.05). The irrigated income per acre (total farming income/total land irrigated) increased ten-fold (p < 0.05). In terms of loan repayment, all participants paid the 600 Ksh down payment for the pump and commodities. Fifty percent of the farmers (9 (41%) of 22 men, and 7 (87%) of 8 women) made the first interim payment and 25% made a 2^nd ^interim payment. Fourteen (93%) of the farmers who made the first payment had an increased family income at the 12-month period. A single farmer who reported a loss of total family income still made a payment. This farmer had an increased irrigated income per acre attributed to pump usage, but reported a loss of other income at the time of survey. Fifty percent of the participants failed to make a single payment.

### Qualitative Experience

Three focus groups were conducted, one with women and two with men. Eighteen of the farmers participated in a focus group discussion. The remaining farmers were not reachable at the time of the focus groups, were not currently in the area, or were too ill to attend. The majority of participants felt that the *Shamba Maisha *program had helped them. Most mentioned that the program provided increased food for consumption in the home and modest improvement in income. As one participant explained, "you can get something to eat and something to sell." A few farmers discussed the investments they had made using money from the program, including buying more land and livestock. Participants also mentioned the benefits of the project more abstractly, saying, "the program...has raised us somehow." One participant described it:

"I felt it was good because if someone gives you something, it gives you some strength to start moving forward and go beyond the level you were doing it."

The most commonly cited challenge to the project was the ongoing drought in the Nyanza province area. A number of farmers had done well at the beginning of the program but had then been hit by the regional drought, making it difficult to repay loans or further invest in their farms. A few farmers discussed limitations of the pump itself, particularly that it did not allow irrigation of a large enough piece of land.

While we did not conduct a quantitative gender analysis, focus group discussions suggested a particular benefit to female participants. Women were more likely to report a change from a state of extreme need to a degree of self-sufficiency. One woman put it this way:

"So the orphans have been eating well because when I pluck the vegetables and someone comes with ten shillings then I can go and buy cooking fat. I can get some more and I go and buy maize flour and now I live with the orphans and they eat." Both men and women expressed an intention to repay the loan, although women were more likely to cite it as one of their economic priorities. Participants in all three focus groups stated that the current drought and subsequent poor production on their farms was the primary reason for their failure to repay.

## Discussion

The *Shamba Maisha *pilot project demonstrated the feasibility of enrolling and following HIV-positive farmers in Nyanza Province into a microfinance based agricultural support program and measuring of health and economic indicators. While the intervention led to a significant improvement in family income, repayment of the loan was poor. Nevertheless, this intervention improved agricultural output, which has the potential to alleviate severe poverty [19].

We successfully tested the feasibility of collecting BMI and CD4 counts and found that these variables could be collected in this population. While there was a trend towards improvement in CD4 counts for those below 200 cells/mcL at baseline, no conclusion can be made as to the effect of the intervention versus the effect of antiretroviral therapy.

Despite the increase in income, multiple unforeseen economic, environmental, and social factors may have impacted the results of our pilot project. During the time period of the study, between June of 2007 and June of 2008, Kenya's economy suffered from rising inflation and the post election violence that occurred following the disputed general election in January, 2008. The inflation rate rose from 5.2% in June of 2007 to 11.2% in June of 2008 [9] and the UN reported that greater than 250,000 people had been displaced by the riots [20]. Furthermore, Kenya experienced a drought during late 2007 and early 2008. In the Kisumu area, farmers did not get the rains they expected between December and March to water their crops and refill water sources. The intervention was designed to allow farming year-round, however, three of the farmers had been using temporary water sources which dried up in the first six months of pump usage causing decreases in total income.

Not unlike numerous other economic empowerment programs [21] the most prominent challenge regarding the feasibility was repayment of the loans. Although the study revealed improvement in income, repayment rates remained low in general. Interestingly, female participants appeared more likely to start paying off the loans, and held repayment of the loans a greater priority than men. Apart from the unforeseen economic, environmental and social factors mentioned above, other possible explanations include use of increased income by farmers to cover other expenses or inadequacy of the loan structure to provide incentives for repayment. While not uncommon, other microfinance schemes have overcome these initial obstacles through group lending and social collateral, placing emphasis on savings accounts, and implementing progressive lending programs [21,22].

Our study had several limitations including the small sample size, cross-sectional design and lack of a control group. The majority of the participants were taking ART, and without a comparable control group the study was not designed to determine the effect of the intervention on health outcomes. These limitations prevent us from making conclusive statements regarding impacts related to the intervention and the study was not powered to detect differences in BMI or CD4 counts. Nevertheless, this pilot was not designed to demonstrate efficacy, but rather feasibility of the intervention and evaluation of health and economic indicators. Expansion of this program will likely require partnership with a local microfinance organization [23] to establish a more rigid economic structure to ensure better repayment. Another limitation of this program was the disproportionate number of female participants. While this was unintentional, it is an important area for consideration. As we know from previous studies, women's economic success is closely tied to household health and well-being [24,25]. Because we were unable to conduct a gendered analysis of the current program due to the small sample size, we cannot assess whether similar gender differences exist for participants of *Shamba Maisha*. Future studies of similar interventions should attempt to recruit a larger group of women, and address the role of gender in the study design.

## Conclusion

The microfinance/micro-irrigation model presented here demonstrates the feasibility of an income-generating micro-irrigation intervention among HIV-positive patients and the collection of health and economic data. Shamba Maisha represents a novel approach to integrating income generating activities and HIV/AIDS programming amongst rural poor farmers. Recent studies have shown the positive impact of microfinance programs on HIV prevention, by reducing risky behaviors and providing opportunities for women's empowerment [16,26]. The connection between food insecurity, poverty and poor health outcomes are well established [27]. Indeed, the Centers for Disease Control and Prevention (CDC) recently recommended using micro-enterprise as a public health measure to combat the HIV/AIDS epidemic among African American women living in the rural South in the United States [26]. Few studies, however, have looked at integrating microfinance and agricultural support programs into the treatment of HIV/AIDS. In addition, as more people living with HIV/AIDS are started on ART and begin living healthy lives, they will increasingly resume their place in society as contributors to the economy. Therefore, further exploration of the health and economic benefits of such interventions coupled with an improved microfinance component may demonstrate a novel approach to economic development for HIV-infected farmers in rural Africa.

## Competing interests

The authors declare that they have no competing interests.

## Authors' contributions

JP assisted in protocol design, carried out on-site recruitment and follow-up, data analysis, manuscript writing and revisions. NS assisted with manuscript writing, editing and revisions. RT carried out focus group discussions and assisted with manuscript writing, editing and revisions. EO carried out on-site recruitment, farmer training, focus group discussions, follow-up, assisted with data collection, interim summaries and communication with the participants. EB oversaw on-site activities, and assisted in protocol and manuscript development, editing and revisions. CC oversaw the all project activities and provided the project with direction, guidance on protocol and manuscript development, editing and revisions. All authors read and approved the final manuscript.

## Pre-publication history

The pre-publication history for this paper can be accessed here:

http://www.biomedcentral.com/1471-2458/10/245/prepub
